# Building a Framework for Sexual and Reproductive Health Care in the Rheumatology Context: Content and Approaches

**DOI:** 10.1002/acr.80049

**Published:** 2026-04-24

**Authors:** Mehret Birru Talabi, Sonya Borrero

**Affiliations:** ^1^ University of Pittsburgh Pittsburgh Pennsylvania

## Abstract

**People with systemic autoimmune and rheumatic diseases (SARDs) are at higher risk than the general population of experiencing adverse pregnancy and perinatal outcomes such as preeclampsia, intrauterine growth restriction, and maternal and/or fetal death. The immune system undergoes changes during pregnancy that can precipitate adverse health outcomes among people with immunologic dysfunction at baseline—particularly those people who have active inflammatory disease at the time of and/or during pregnancy. However, in this commentary, we challenge the notion that immunologic and disease‐centric aspects of pregnancy are the only drivers of adverse pregnancy and perinatal outcomes among people with SARDs. A focus on disease may have limited focus on other factors that could enhance reproductive health outcomes among people with SARDs, for example, the provision of high‐quality sexual and reproductive health (SRH) care in the rheumatology context. Identification of such upstream factors can reveal approaches that enhance downstream reproductive outcomes for all women with rheumatic diseases, thereby enhancing person centeredness, holistic care, and SRH equity in rheumatology care. Herein, we also describe emerging approaches to SRH that provide a potential framework for high‐quality SRH care in the rheumatology context**.

## Introduction

Sexual and reproductive health (SRH) is an important aspect of overall health for individuals with systemic autoimmune and rheumatic diseases (SARDs).^1^ The majority of people with autoimmune conditions are female.^2^ The hormonal and immunologic changes of pregnancy can accelerate disease progression and contribute to adverse maternal and fetal outcomes among people with SARDs, particularly in systemic lupus erythematosus (SLE) and antiphospholipid syndrome (APS).^3^, ^4^, ^5^ One to two percent of pregnancies among women with Ro antibody positivity will be complicated by fetal cardiac conduction abnormalities that may lead to fetal death or require lifelong cardiac pacing.^6^ Even pregnancies among women with rheumatoid arthritis (RA)—a condition that may symptomatically improve during pregnancy—are associated with higher risk of preeclampsia and fetal growth restriction compared to the general population.^7^ In general, active disease at the time of pregnancy and/or during pregnancy is a key risk factor for adverse pregnancy and perinatal outcomes such as mortality and severe maternal morbidity.^8^

The disease‐modifying antirheumatic drugs (DMARDs) used to control SARDs may also threaten healthy pregnancies. Although some treatments are safe for use during pregnancy and lactation, many others have unknown safety profiles, and several treatments have teratogenic potential. In a study of the American College of Rheumatology (ACR) Rheumatology Informatics System for Effectiveness registry, which captures clinical data from approximately one‐third of US rheumatology practices, 44.5% of nonpregnant young females with RA and 29.5% with SLE were prescribed at least one teratogen; descriptive data confirm that some people become pregnant while prescribed these drugs.^9^, ^10^ Many teratogens cause irreversible anomalies in the first trimester of pregnancy—a point at which some women are unaware that they are pregnant.^11^ Contraception usage among women with SARDs appears to be lower than that of the general population—even when teratogens are prescribed—possibly due to concerns about the safety of exogenous hormones in this population.^12^

However, adverse reproductive outcomes are not inevitable across the SARDs; many women with SARDs experience healthy pregnancies and reproductive lives. Across disease states, a key predictor of healthy SRH outcomes is well‐controlled disease for at least three to six months before pregnancy.^13^, ^14^, ^15^ As advances in treatment are leading to reductions in disease burden and organ damage, individuals with SARDs may increasingly enter pregnancy with better baseline health that can facilitate favorable downstream pregnancy outcomes.

**Figure 1:**
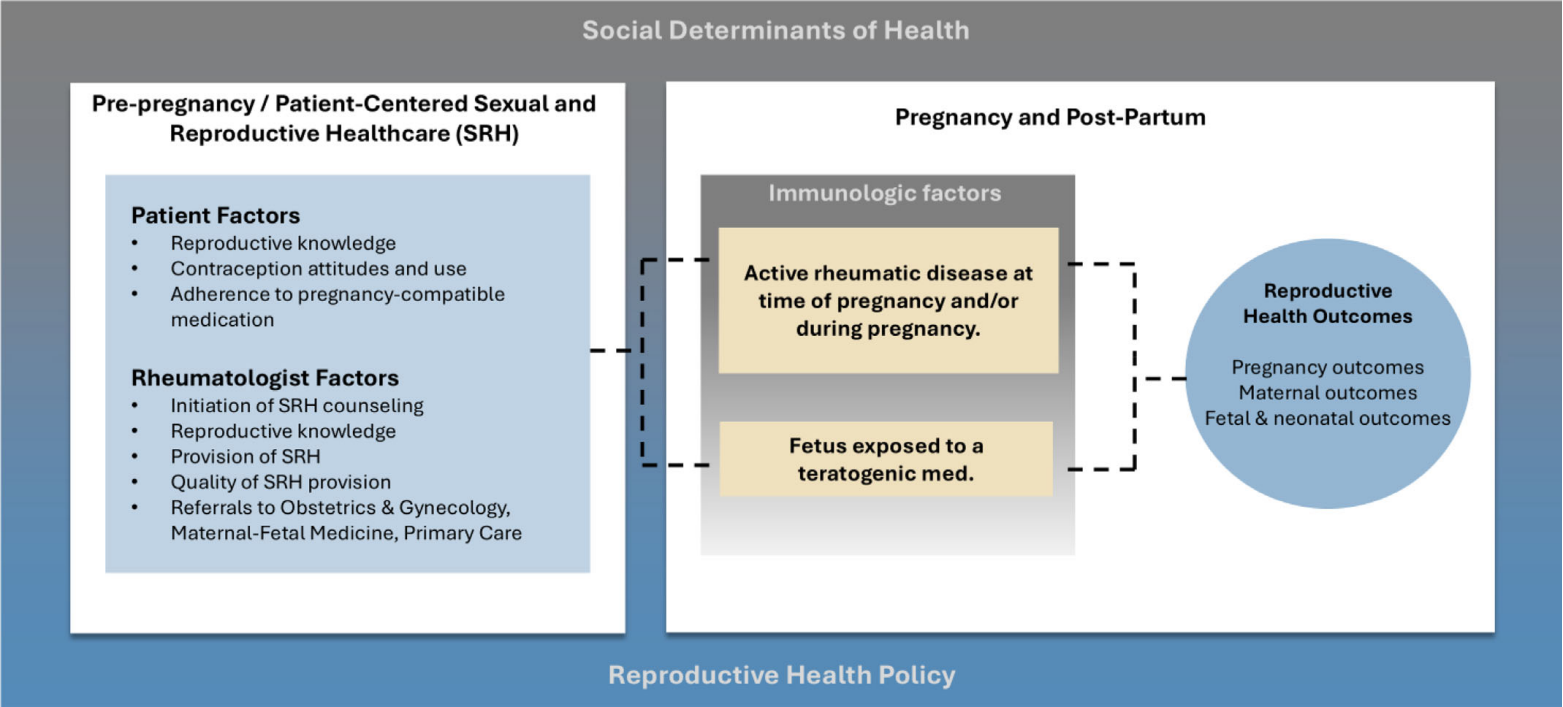
Novel conceptual model describing factors that contribute to sexual and reproductive health outcomes among individuals with systemic autoimmune and rheumatic diseases.

However, immunologic factors provide an incomplete explanation for why some women experience healthy or unhealthy pregnancy outcomes (Figure 1). For example, social determinants of health, including income, education, health insurance, and transportation, affect people's access to health care during the perinatal period.^16^ Clinical outcomes such as severe maternal morbidity and mortality among people with SARDs are rarely reported in the context of state‐ or federal‐level reproductive health policies; however, such policies affect access to preventive and therapeutic SRH services (eg, prenatal care, pregnancy and antepartum care, contraception, abortion care).^17^

An oft‐overlooked factor that also influences reproductive outcomes for people with SARDs is the quality and availability of SRH care.^18^ SRH care encompasses a range of services that help people to safely attain their desired number of children (if any), determine the spacing and timing of pregnancies, learn about and obtain contraception if needed, and obtain relevant preventive services (eg, cervical cancer screening). Rheumatologists who assess patients’ pregnancy preferences can help to prepare people for a desired pregnancy, mitigate risk factors for adverse pregnancy outcomes, prescribe pregnancy‐compatible medications, and refer to primary care providers (PCPs), obstetrician–gynecologists (OB/GYNs), reproductive endocrinologists, and potentially other specialists to optimize prepregnancy health. Rheumatologists can also counsel peripartum patients about pregnancy‐compatible treatments in lactation and manage postpartum disease activity. Moreover, rheumatologists can help people who wish to avoid or delay pregnancy to identify safe contraceptive methods, thus preventing pregnancies that are undesired and potentially high risk.

Given a rheumatology workforce shortage and widespread burnout,^19^ some rheumatologists might argue that SRH care for patients with SARDs should largely be delegated to PCPs or OB/GYNs—currently a dominant model of care. A counterargument is that by diverting most SRH care and counseling to nonexperts in the SARDs, our field misses a critical chance to improve reproductive outcomes for patients at the core of our professional mission. Rheumatologists with specialized experience and interest in SRH (ie, “reproductive rheumatologists”) contribute to research, education, and patient care at the intersection of rheumatology and SRH. However, SRH care should not be a niche competency in rheumatology. To ensure that all patients with SARDs equitably receive high‐quality SRH care and thus have the opportunity to experience their healthiest reproductive lives, practical and robust standards for the provision of this care must be developed in rheumatology.

## A new opportunity to reconceptualize SRH care in rheumatology

Now is a particularly opportune time to reconceptualize how SRH can be meaningfully addressed in rheumatology care. Other subspecialties are similarly deliberating the minimum competencies that clinicians should have in SRH care provision^20^; for example, cardiologists are advancing “cardio‐obstetric” training and multidisciplinary care models to ameliorate the cardiovascular disease burden that underlies most maternal deaths.^21^ Broadly, the US crisis of maternal morbidity and mortality has led experts to interrogate the health care system and challenge long‐standing models of SRH care.^22^ For example, the once ubiquitous paradigm of “family planning” has increasingly fallen out of favor, as described in the following:

- A high proportion of pregnancies in the United States remain unintended, despite a century of public health approaches to promote pregnancy planning.^23^
- Family planning praxis was developed in the backdrop of the eugenics movement, and forced sterilization and contraception policies directed against racial and ethnic minority groups, poor people, and individuals with disabilities have caused long‐standing harms and, understandably, medical distrust in some communities.^24^
- Planning for a family may personally resonate for clinicians,^25^ but may not reflect the lived realities of other people.
- Metrics around unintended pregnancy are shifting toward those that evaluate an individual's acceptance of a pregnancy, as an unintended but welcomed pregnancy can still motivate healthy behaviors and lead to positive clinical outcomes.^26^
- Many women have fluctuating, ambivalent, conflicted, or fatalistic attitudes around pregnancy; clinical approaches that require these individuals to report a discrete pregnancy decision may inaccurately assess their potential for pregnancy.^27^
- Public health measures to reduce unintended pregnancy may have inadvertently led to clinical approaches that guide or pressure people to align their reproductive behaviors with public health objectives.^27^

In contrast, emerging health care approaches are centering personalized, holistic, autonomy‐supportive SRH care—framed, for example, by the praxis of reproductive justice, which indicates that people have a “(human) right to have a child, not to have a child, and to have safe and healthy conditions under which their children may be raised.”^24^ Through person‐centered approaches, clinicians tailor their counseling to individuals based on their priorities and life circumstances—meeting people where they are—rather than requiring or attempting to persuade an individual to tailor their needs toward a specific approach (eg, planning for pregnancy) or outcome (eg, pregnancy avoidance).^28^ Person‐centered SRH care in rheumatology may appear to conflict with data indicating that advance planning for pregnancy helps to ensure disease control while taking pregnancy‐safe medications. Indeed, although pregnancy planning is reasonable for patients with serious chronic illnesses that can impact pregnancy outcomes and will even be appealing to some patients, unplanned pregnancies, by active or passive choice, will remain a reality for some patients.^28^ A model of SRH care is needed in rheumatology that addresses all patients’ SRH needs, including individuals whose preferences and actions do not align with planning paradigms or medical advice.

Recent federal guidance provides a patient‐centered SRH care model for consideration for patients with SARDs. In 2024, the US Office of Population Affairs updated its Quality Family Planning (QFP) Services guidelines, a series of expert‐ and evidence‐driven recommendations to ensure that all people of reproductive age receive high‐quality SRH services.^29^ The updated QFP provides guidance for any clinician who sees patients with SRH care needs, including those who are the primary providers of SRH services (eg, OB/GYNs, PCPs) and those who are not; rheumatology and other subspecialties are explicitly mentioned in this context. The QFP asserts that these clinicians should “screen for the need and desire for SRH services, share the limits of their practice, and refer or connect patients to other settings where they can obtain quality services.” The QFP aligns with the 2020 ACR “Guideline for the Management of Reproductive Health in Rheumatic and Musculoskeletal Diseases” (colloquially called the “Reproductive Health Guideline” or RHG), which recommends that SRH conversations between the rheumatologist and patient occur early and often, “at an initial or early visit and periodically thereafter, and always when initiating treatment with potentially teratogenic medications.”^1^

In the following sections, we build from QFP and ACR guidance to propose core competencies and approaches for a basic standard of SRH care in rheumatology (Box 1). Our recommendations are not definitive or comprehensive and are meant to stimulate discussion about ways that SRH care can be meaningfully integrated into rheumatology practice. Recommendations reflect the authors’ opinions and do not reflect the official policy or positions of the ACR.

Box 1 **Suggestions for how to build sexual and reproductive health (SRH) care competencies in rheumatology care**

**Build core competencies in SRH for all rheumatologists**

- Use resources from the American College of Rheumatology (ACR),^1^ EULAR,^13^, ^47^ and other professional societies that provide comprehensive recommendations for management of the patient with SRH needs.
- Rheumatology, obstetrics and gynecology, and/or maternal–fetal medicine professional organizations should codevelop continuing medical education tailored to rheumatology clinicians.
- SRH didactics should be available at regional and national rheumatology meetings, ideally scheduled at times to optimize participation.
- Rheumatologists should build competency in prescribing or recommending over‐the‐counter progestin‐only pills and/or emergency contraception to people who desire contraception and have an urgent need (eg, at initiation of a teratogenic medication).
- Rheumatologists should be aware of freely available educational resources around SRH for clinicians and patients:
  - Mother to Baby, which provides online and phone‐based resources in English and Spanish for medication safety in pregnancy and lactation (www.mothertobaby.org)
  - The Healthy Outcomes in Pregnancy with SLE Through Education of Providers (HOP‐STEP) Program, which provides SRH information for patients with systemic lupus erythematosus (SLE) and other systemic autoimmune and rheumatic diseases (SARDs) (www.lupuspregnancy.org)
  - LactMed, an online resource providing information about medication safety in lactation^76^

**Build advanced expertise in SRH among interested rheumatologists**

- A formalized certification program in “reproductive rheumatology” could build a cadre of rheumatology professionals with SRH knowledge and expertise.
  - A formalized certification program in reproductive rheumatology could include professional education materials, digital lectures, an online certification examination and use study materials from existing textbooks and guidelines from ACR, EULAR, and British Society for Rheumatology.^77^, ^78^, ^79^
- Develop pregnancy registries and SRH‐focused rheumatology research registries to generate data advancing SRH care.
- National meetings of experts in rheumatology and trainees/early‐stage investigators interested in reproductive rheumatology to discuss emerging findings in the field and challenging cases and build cross‐institutional collaborations.
  - Examples include the Reproductive Rheumatology National Lab Meeting monthly teleconference funded by the National Institute of Arthritis and Musculoskeletal and Skin Diseases, NIH (Duke University, Principal Investigator: Megan E.B. Clowse, MD MPH).
- International SRH‐focused rheumatology meetings, such as the International Conference on Reproduction, Pregnancy, and Rheumatic Diseases (“Rheumapreg”), highlight multidisciplinary SRH care models for patients with SARDs.

## SRH care in rheumatology: Core competencies for the rheumatologist

**Figure 2:**
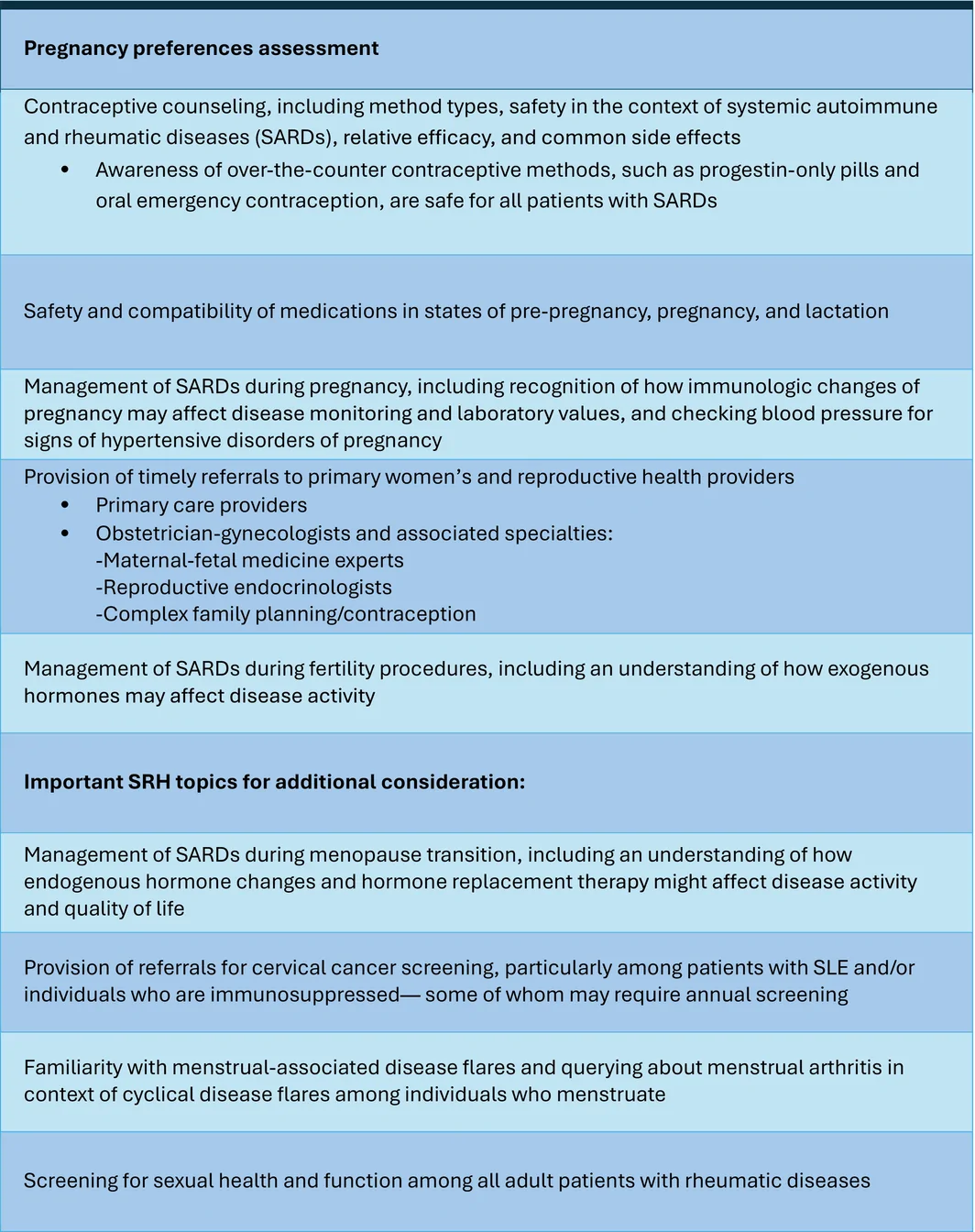
Recommended sexual and reproductive health (SRH) competencies for rheumatologists by topic area. SLE, systemic lupus erythematosus. Color figure can be viewed in the online issue, which is available at http://onlinelibrary.wiley.com/doi/10.1002/acr.80049/abstract.

Although all rheumatologists should have at least a basic understanding of SRH care in the rheumatology context, the degree of requisite education and training will vary by the rheumatologist's interests, prior experience and training, patient population, and scope of practice. Herein and in Figure 2, we suggest core SRH competencies and knowledge domains for the practicing rheumatologist. Box 1 presents the following: (1) ways to potentially build these core competencies and (2) opportunities for rheumatologists who seek advanced clinical training, scholarship, and/or professional demonstration of SRH competency.

### Assessment of pregnancy preferences

Rheumatologists should feel comfortable with initiating conversations about SRH with patients. Screening for reproductive preferences increases the frequency of counseling as well as patient satisfaction.^30^, ^31^ Around the time of diagnosis, we recommend that rheumatologists explain to patients that reproductive health is integral to overall health in the SARDs and encourage patients to raise SRH concerns or questions during and/or between visits. Although there is no consensus on the frequency with which pregnancy preferences should be assessed, at a minimum, we recommend doing so annually and, as per the ACR RHG, when teratogenic medications are considered or prescribed.^1^ The QFP further suggests that clinicians should ask patients to openly communicate their reproductive goals, preferences, and priorities.^29^ The clinician next should, without judgment, share personalized and general information about these goals and preferences and then, together with the patient, tailor the care plan to help to ensure that the patient's needs are met as safely as possible.^32^ The QFP also indicates that the clinician should “confirm understanding” about information conveyed, as some people may not recall or understand the information that is offered during the clinic visit.^29^

Simple and open‐ended questions can provide a respectful foundation for SRH conversations. One Key Question (OKQ) could be used to elicit patients’ pregnancy preferences: “Would you like to become pregnant in the next year?” When tested in rheumatology clinics as a quality improvement (QI) initiative, OKQ appeared to increase referrals for OB/GYN care, although it had poor uptake into clinical practice.^33^ Critiques of OKQ include that it does not capture patients’ attitudes around pregnancy prevention and focuses on a limited time frame (ie, one year). In contrast, the Pregnancy Attitudes, Timing, and How Important Is Prevention (PATH) questions help to elicit patients’ orientations around pregnancy and contraception through three questions: (1) “Do you think you might like to have (more) children at some point?” (2) “When do you think that might be?” and (3) “How important is it to you to prevent pregnancy (until then)?“^27^ These questions guided appropriate SRH referrals in the general population.^34^ For patients with SARDs, we recommend additional language to gauge a patient's understanding of their health risks in the SRH context: (1) “I hear your reproductive goal is (x). Did I get that right? I want to help you to meet your goals as safely as possible.” (2) “What is your understanding of how your (disease) might impact your (reproductive goal or preference)?” (3) “Can I share with you what I know about your (disease/medications) and how it might affect your (reproductive goal or preference)?“^18^

As rheumatologists engage in SRH care, we advocate for an approach framed by cultural humility, in which the clinician respectfully considers social, historical, and/or lived experiences that might affect an individual's preferences and priorities apart from disease control.^35^ Such contexts might include a person's religious beliefs, cultural preferences, and/or an acknowledgment of health care traumas perpetuated on historically marginalized populations.

### Pregnancy and fertility care

Once rheumatologists have assessed people's preferences for pregnancy, rheumatologists may advise women who desire pregnancy to take a prenatal vitamin and/or folic acid—particularly if they have used a medication that can reduce folic acid stores (eg, methotrexate, sulfasalazine). Disease activity and medication safety and compatibility in pregnancy should ideally be assessed before pregnancy. Rheumatologists should strongly consider prescribing hydroxychloroquine to patients with SLE and with high‐titer Ro/SS‐A antibodies to prevent neonatal lupus and fetal heart block.^1^ During pregnancy, the ACR RHG recommends that disease activity laboratory tests are checked at least once a trimester.^1^ Rheumatologists should be aware that the biologic changes of pregnancy can affect the assessment of disease activity. For example, during normal pregnancy, complement levels rise by 10% to 50%, physiologic proteinuria of less than 300 mg/day is common, the erythrocyte sedimentation rate can increase by 30% to 70%, and increases in intravascular volume can lead to mild anemia and thrombocytopenia.^36^ Rheumatologists should also be aware that as compared to the general population, some patients with SARDs—particularly SLE and APS–are at higher risk for potentially life‐altering hypertensive disorders of pregnancy (eg, preeclampsia, HELLP [hemolysis, elevated liver enzymes, and low platelets] syndrome).^37^ Low‐dose aspirin (LDASA), started by the end of the first trimester, may modestly reduce the risk of preeclampsia among individuals with SLE, antiphospholipid antibodies (aPLs)/APS, and other risk factors for preeclampsia (eg, chronic hypertension, history of preeclampsia); whereas OB/GYNs usually initiate LDASA as part of their practice, rheumatologists can also safely do so.^1^, ^38^, ^39^ Rheumatologists who see pregnant patients whose blood pressures meet or exceed systolic and diastolic pressures of 140 mm Hg and/or 90 mm Hg, respectively, should directly communicate with OB/GYN colleagues, as urgent treatment and monitoring may be required.

Fertility is generally preserved in the SARDs; however, some patients may experience subfertility or infertility related to cyclophosphamide exposure, uncontrolled RA, or advanced maternal age.^1^ Rheumatologists should promptly refer patients with suspected infertility to reproductive endocrinology for evaluation and consideration of assisted reproductive technologies (ART).^40^ Patients with stable or quiescent diseases, including SLE, can safely undergo ART; however, during ovarian stimulation, low molecular weight heparin should be considered for individuals with aPLs and provided to individuals with obstetric or thrombotic APS.^1^ To preserve reproductive potential for women using intravenous cyclophosphamide, reproductive endocrinologists can facilitate oocyte preservation before therapy, and rheumatologists or OB/GYNs may consider prescribing gonadotropin‐releasing hormone agonist therapy for ovarian protection (eg, leuprolide acetate). With the exception of cyclophosphamide, all other immunosuppressive therapies—including those with teratogenic risk—can be continued during ovarian stimulation for oocyte/embryo cryopreservation; teratogens, however, must be discontinued in advance of implantation and during pregnancy.^1^

### Medication safety during pregnancy and lactation

DMARDs are often required before, during, and after pregnancy to control SARDs disease activity and thus may be essential for optimizing downstream reproductive outcomes.^41^ However, despite revisions to the Federal Policy for the Protection of Human Subjects—the “Common Rule”—which declassified pregnant women as a protected class in research in 2019, many drug and vaccine trials continue to exclude pregnant women. Rather than enhancing safety in this group, this approach to research has undermined the generation of evidence needed to inform safe prescribing decisions during pregnancy and lactation.^41^, ^42^ Among newer drugs approved by the US Food and Drug Administration (FDA), 90% present limited or no human data on pregnancy safety.^43^ More research is urgently needed to investigate the safety of medications in the pregnancy and lactation context and to determine how potential risks can be clearly communicated to clinicians and patients.

All rheumatologists should be aware of evolving guidance around medication safety in pregnancy and lactation. The ACR RHG and EULAR indicate that certain medications are not safe to use in pregnancy, including cyclophosphamide (in the first trimester), methotrexate, and mycophenolate/mycophenolic acid.^1^, ^13^ Although azathioprine is safe to use throughout pregnancy, the FDA has issued a warning around a rare association of thiopurines with intrahepatic cholestasis of pregnancy (ICP). One study of SLE reported a 4.7% risk of ICP in thiopurine‐exposed pregnancies versus 1.9% in unexposed pregnancies. This complication resolves with drug discontinuation and may potentially be mitigated by twice‐daily dosing.^44^, ^45^ The FDA has also advised against use of nonsteroidal anti‐inflammatory drugs beginning around 20 weeks’ gestation due to risk of fetal oligohydramnios and at around 30 weeks’ gestation due to risk of premature closure of the fetal ductus arteriosus.^46^ Other medications, such as hydroxychloroquine, sulfasalazine, colchicine, tumor necrosis factor inhibitors, and low‐dose steroids, are safe to use at all stages of pregnancy.^1^, ^13^

EULAR also asserts that rheumatologists may also consider treating patients with biologic drugs with molecular properties that render them unlikely to cross the placenta during early fetal development.^13^ For example, monoclonal antibodies of IgG (eg, belimumab, canakinumab, ixekizumab, rituximab, sarilumab, secukinumab, tocilizumab, ustekinumab, rituximab) have high affinity to neonatal Fc receptor (FcRn) but are unlikely to cross the placenta until after 16 weeks’ gestation, a point after which fetal organogenesis has concluded and risk of teratogenesis is low. The FcRn has weak binding affinity to the Fc fusion proteins of certain biologics (eg, etanercept, abatacept), and neonatal drug levels are low. Fc‐free drugs and biologic drugs without Ig structures (eg, certolizumab, anakinra) do not enter the fetal circulation.^13^ This guidance significantly broadens the therapeutic armamentarium for people who require treatment during pregnancy and thus may positively impact pregnancy outcomes in the SARDs.

Following a case of disseminated tuberculosis in an infliximab‐exposed infant who received the live BCG vaccine, a number of studies examined the health risks associated with fetal exposure to biologics and have not found an increased risk of major infections among these children in their first year of life.^47^ ACR and EULAR indicate that nonlive vaccines are safe to give on schedule to biologic‐exposed infants, and the live rotavirus vaccine can be given on schedule to infants exposed to some biologics (eg, tumor necrosis factor inhibitors). At present, guidelines conditionally recommend delay of the rotavirus vaccination until six months of age for infants exposed in utero to anti–B cell agents (eg, rituximab, belimumab) and potentially other biologics that are less well studied.^13^, ^48^ All guidelines indicate that risks of neonatal immunosuppression with in utero exposure to DMARDs must be balanced with the potential consequences of uncontrolled disease activity on the patient and fetus.^1^, ^13^, ^49^

### Contraception

All people with SARDs can use at least one method of contraception safely; however, OB/GYNs and PCPs may be less familiar with safety issues related to the SARDs. We therefore believe that basic contraception counseling and care is important for rheumatologists to build into their clinical practice. Clear contraception safety guidance is available from the ACR RHG and the Medical Eligibility Criteria from the Centers for Disease Control and Prevention.^1^, ^50^ Methods that appear to be safe for nearly all patients, including those with SLE and moderate‐ or high‐titer aPLs/APS, are the intrauterine device (IUD), emergency contraception (including over‐the‐counter oral tablets or the copper IUD, which is hormone free), and the progestin‐only pill (POP).^1^, ^51^, ^52^, ^53^

Underscoring the safety of POPs, an FDA‐approved POP, norgestrel, has been available for purchase over the counter since 2023.^54^ Rheumatologists should feel comfortable recommending this option to patients, particularly those with an urgent contraceptive need and with appropriate referrals to OB/GYN or contraception specialists for follow‐up care. Importantly, norgestrel currently costs around $20 for a one‐month supply, which will be cost‐prohibitive to some patients; rheumatologists ideally would feel comfortable prescribing POPs, which are low‐cost or even free with certain insurance plans.

### Menstruation, menopause, and sexual function

The rheumatologist should be generally aware of how menstruation and menopause may affect SARDs activity and quality of life. Although understudied, menstrual arthritis—disease flares occurring around the time of menstruation—can be debilitating and, if not explicitly screened for among menstruating people, can be misconstrued as treatment‐resistant disease activity. In up to half of cases, contraception and/or nonsteroidal anti‐inflammatory drugs may ameliorate these symptoms without the need for DMARD escalation.^55^ The menopause transition is associated with emergence of SARDs such as RA and Sjögren disease and can be difficult to differentiate from the recently characterized “musculoskeletal syndrome of menopause,” which includes perimenopausal/menopausal arthralgia, osteoarthritis, adhesive capsulitis, tendinitis, osteoporosis, sarcopenia, and/or diffuse musculoskeletal pain.^56^, ^57^ During menopause, sexual dysfunction becomes more prevalent among women with vasomotor and genitourinary symptoms, the latter of which may be particularly challenging for women with pre‐existing vaginal dryness from SARDs (eg, Sjögren disease).^58^

Vasomotor, genitourinary, and potentially even musculoskeletal symptoms may improve with systemic or topical estrogens and/or progesterone, ie, menopausal hormone therapy (MHT), previously called hormone replacement therapy. Recently reinterpreted clinical data suggest that MHT is safer than previously recognized for many women younger than 60 years and/or within 10 years of menstrual cessation^59^; midlife patients may increasingly ask their rheumatologists about the safety of MHT in the context of their SARDs. Of note, MHT formulations have lower estrogen content than estrogen‐containing contraceptives.^60^ The ACR RHG indicates MHT—particularly transdermal options (eg, patch), which have less of a thrombotic risk than oral options—can be safely considered for individuals with SLE and other SARDs. MHT currently is not recommended for individuals with APS or conditionally for individuals with persistently positive aPLs, as the acceptably safe dose of exogenous estrogens in this group is unknown.^1^ Individuals with genitourinary symptoms who are not candidates for MHT can use vaginal estrogen, which has minimal systemic absorption. For vasomotor symptoms, these individuals can consider FDA‐approved paroxetine, a selective serotonin reuptake inhibitor (SSRI), or the expensive but effective neurokinin receptor antagonists (NK1/3, NK3), which modulate thermoregulatory zones in the hypothalamus. Nonhormonal, off‐label options for vasomotor symptoms include nonparoxetine SSRIs, serotonin–norepinephrine reuptake inhibitors, gabapentin or pregabalin, oxybutynin, and cognitive behavioral therapy.^60^

## Building structural resources that enhance SRH care for patients with SARDs

A challenge for the rheumatologist is how to operationalize SRH competencies within existing clinical care models. Qualitative findings and QI projects provide guidance for the integration of SRH into practice.^33^, ^61^, ^62^, ^63^ However, the creation of an implementable SRH framework in rheumatology will also require multidisciplinary clinical collaboration, trainee education, and patient information resources, as described in Box 2 and the following sections:

Box 2 **Building structural resources that enhance sexual and reproductive health (SRH) care for patients with systemic autoimmune and rheumatic diseases (SARDs)**

**Build collaborative care models between rheumatologists, reproductive rheumatologists, and obstetrician–gynecologists (OB/GYNs)/maternal–fetal medicine (MFM)**

- Identify an OB/GYN practice or OB/GYN with whom to collaborate on SRH care.
- Develop multidisciplinary case conferences at academic centers that highlight clinical pearls.
- Use the electronic health record to do the following:
  - Create easy referral pathways between OB/GYN and rheumatology.
  - Collect local data about SRH outcomes; gaps in care could be prioritized for quality improvement.
- Develop virtual or electronic cross‐institutional consultation systems between reproductive rheumatologists and community‐based rheumatology providers for challenging SRH cases.^80^
- Develop easy referral pathways between rheumatology and complex contraception specialists—OB/GYN clinicians who provide contraception for people with medical complexity.
- Develop collaborative clinical care models between OB/GYN and rheumatology, such as the following:
  - Reproductive rheumatology clinics that provide guidelines‐concordant SRH care and coordinate care with OB/GYN and OB/GYN specialists
  - Current examples include the University of Pittsburgh/University of Pittsburgh Medical Center, Duke University, Hospital for Special Surgery, and University of Chicago.
  - Rheumatology–obstetric clinics that integrate rheumatology and OB/GYN care
  - US example includes Women & Infants Hospital in Providence, Rhode Island^81^; multiple clinics internationally offer this care in a single, combined clinic.^82^
  - As 30% of maternal deaths occur 42 days after delivery until one year post partum, Interconception Care (ICC) and Fourth Trimester (4TM) care models engage primary care clinicians and subspecialists in management of maternal health immediately after and between pregnancies to enhance postpartum and future pregnancy outcomes.^83^, ^84^

- Develop large‐scale research centers that integrate SRH and rheumatology/chronic disease management and advance the evidence base for SRH care for individuals with SARDs:
  - U.S. examples include University of Pittsburgh,^83^ Duke University, Hospital for Special Surgery, and New York University.

**Build SRH education for rheumatology trainees**

**National or regional level**

- Perform an updated needs assessment around SRH education in rheumatology.^70^
  - Include rheumatology advanced practice providers.
- Develop standards for SRH education in rheumatology fellowship, developed by content experts in reproductive rheumatology, medical education, and women's health specialties.
- Increase representation of SRH topics on the rheumatology initial certification examination.
- Increase awareness of and access to educational opportunities to build SRH knowledge:
  - Expand access to up‐to‐date lectures (eg, American College of Rheumatology [ACR] Virtual Rheumatology Program for Fellows in Training); provide pre‐ and post‐tests to assess knowledge acquisition.
  - Require continuing medical education on SRH topics.
- The Objective Structured Clinical Examination can assess competency in counseling around contraception or pregnancy planning and can be proctored virtually or in person by content experts in reproductive rheumatology or OB/GYN/women's health.^85^
- A certification program in rheumatology and SRH could build expertise in reproductive rheumatology.
- Increase research opportunities for interested fellows in topics related to SRH.
  - Cross‐institutional collaborations may be needed if local expertise is not available.
- Foundation‐ and industry‐funded one‐year fellowships at reproductive rheumatology centers could advance a cadre of highly trained rheumatologists in SRH (eg, UCB Women's Health Fellowship in Rheumatology).

**Institutional level**

- Develop local SRH curricula and training opportunities (eg, clinic rotations) with faculty from OB/GYN/MFM, reproductive endocrinology, and complex contraception.
- Invite conference speakers with clinical and research expertise in SRH (ie, reproductive rheumatologists) to lecture to trainees and faculty.
- Implement multidisciplinary case‐based conferences for training programs that lack access to patients who have SRH/obstetric needs based on population demographics with OB/GYN/MFM experts to review clinical scenarios that they may face in practice.
- Routinely observe and evaluate fellows while providing an aspect of SRH care and counseling to patients.
- Encourage trainees to develop and implement quality improvement projects focused on SRH, which have measurable outcomes and can augment SRH education and quality of care within a single rheumatology division.

**Develop patient‐directed resources focused on SRH and SARDs**

- ACR, Arthritis Foundation, Lupus Foundation of America, Vasculitis Foundation, Mother to Baby (www.mothertobaby.org), HOP‐STEP (www.lupuspregnancy.org), and Bedsider (www.bedsider.org), among others, offer person‐centered SRH materials online.
- SRH materials in the SARDs context must be accessible to individuals with limited health literacy and numeracy skills.
- Digital resources can offer SRH information directly to patients:
  - Digital resources have been developed for patients with SARDs.
  - MyVoice:Rheum is a digital decision aid that may build reproductive knowledge among women with SARDs.^74^
- Trained doulas, community health workers, and/or peer support groups, in addition to clinicians, may support information needs, as well as emotional and physical needs related to pregnancy, parenting, pregnancy loss, adoption/surrogacy, or other areas of SRH.

### 1. Build bridges with primary care and obstetrics and gynecology

Although patients need PCPs and OB/GYNs to address SRH needs such as pregnancy care and contraception, OB/GYN shortages, particularly in rural areas and states with abortion restrictions, are undermining access to SRH care.^64^, ^65^ Among nearly 2,500 reproductive‐aged women with SARDs who received regular rheumatology care in a large health care system, only one‐third and two‐thirds saw a gynecologist or PCP, respectively, over a two‐year time frame.^66^ Some patients report that no provider takes ownership of their SRH needs or that their rheumatologists and OB/GYNs provide conflicting advice, particularly around contraception safety or medication safety during pregnancy and lactation.^10^ Moreover, some rheumatologists report that they lack professional relationships with OB/GYNs.^67^

Rheumatologists can no longer safely assume that patients have an SRH provider. Ideally, every rheumatology practice should be able to identify at least one OB/GYN provider or practice to whom they can place referrals for urgent patient care. We believe it is particularly important that rheumatologists and maternal–fetal medicine (MFM) experts—obstetric specialists who care for patients with chronic and complex medical conditions—work collaboratively and provide clinical care from a common evidence base. To better meet the SRH needs of individuals with SARDs, structural changes are urgently needed to break silos and improve communication, collaboration, and guidelines‐concordant care between rheumatologists and OB/GYNs/MFM.

### 2. Build rheumatology fellowship training in SRH

Rheumatologists’ knowledge and comfort level around SRH care may reflect their exposure to SRH in the training pathway. It is unclear how many rheumatology fellowship programs currently integrate SRH into their curricula. SARDs‐specific aspects of women's and SRH care are not explicitly featured in the Accreditation Council for Graduate Medical Education program requirements for rheumatology fellowship training.^68^ The rheumatology certification examination includes content areas such as “lupus pregnancy,” “neonatal lupus,” and “antiphospholipid antibody syndrome in pregnancy.”^69^ However, important aspects of peripartum care across other SARDs, pharmacologic management in pregnancy and lactation, and contraception care do not appear to be featured in the primary content of the examination. It is therefore conceivable that some rheumatologists enter independent practice without having received formal SRH education or demonstrated SRH knowledge. Even less is known about SRH training for advanced practice providers (APPs), who are increasingly providing rheumatology care in the United States.

To build a foundation of SRH in rheumatology, SRH education must be integrated into rheumatology fellowship and APP training; fellows have greater bandwidth to learn about SRH than practicing rheumatologists, and a prior study indicated that they are eager to expand their SRH knowledge using summary sheets, question banks, didactics, and online modules.^70^ To enhance accountability, rheumatology in‐training and certification examinations should cover a broader range of SRH topics. Fellows in training and APPs should also learn how to routinely assess people's reproductive goals and preferences as part of the standard clinical encounter.

### 3. Build evidence‐based educational resources for patients with SARDs

Patient knowledge may be an important driver of downstream SRH outcomes. Some patients lack basic and SARDs‐specific SRH knowledge, such as the effectiveness and safety of contraception in the context of SARDs. Such knowledge gaps might lead some patients to inadvertently choose low‐efficacy methods of contraception, increasing the risk for pregnancies that are undesired and may be high risk.^31^, ^71^, ^72^ In addition, some patients discontinue effective, pregnancy‐compatible medications during pregnancy and lactation due to uncertainty about medication safety, which can lead to suboptimal clinical outcomes.^10^

Patients need trustworthy, accurate, and accessible information about SRH. Rheumatologists and/or support staff (eg, nurses) should be prepared to share educational materials about SRH. As SRH decisions are often nuanced, complex, and preference sensitive, individuals need time to reflect on their own priorities and sometimes seek opinions of their partners or family members; they also need ways to communicate their questions to their rheumatologists. Resources are available to educate patients and help to build their SRH knowledge and clarify and communicate their information needs, such as videos, websites and printable fact sheets,^73^ decision aids,^41^, ^74^ SRH knowledge assessments for in‐clinic and research use,^72^, ^75^ and educational interventions.^75^

## Conclusions

An important opportunity exists to enhance the SRH of individuals with SARDs through proactive care provided in the rheumatology context. We highlight potential targets for intervention before and during pregnancy that may help to support healthy reproductive outcomes that align with people's preferences and priorities. We offer ideas about how existing SRH‐related resources from the QFP, ACR, and EULAR can be integrated into rheumatology care.

We acknowledge that our recommendations have limitations. First, we focus on female patients with SARDs; however, adolescents, male patients, and LGBTQIA individuals also need and deserve high‐quality SRH services and should be the focus of future deliberation. Furthermore, our recommendations are not exhaustive; for example, although not addressed in depth in the current article, referrals for cervical cancer screening and assessments for sexual dysfunction and postpartum depression are important in certain populations. Finally, additional brainstorming is needed to feasibly advance SRH delivery in rheumatology clinics with high patient volumes and/or abbreviated visit times.

However, by building a stronger foundation of SRH care in rheumatology, we can reconceptualize the adverse reproductive outcomes that can occur in rheumatology as being intervenable and not inevitable; this can provide reassurance and hope for patients with SARDs. Moving forward as a field, we must respectfully and proactively deliberate how best to integrate SRH care in rheumatology in ways in which rheumatologists will support and invest and that best meet the needs of individuals with SARDs.

## AUTHOR CONTRIBUTIONS

All authors contributed to at least one of the following manuscript preparation roles: conceptualization AND/OR methodology, software, investigation, formal analysis, data curation, visualization, and validation AND drafting or reviewing/editing the final draft. As corresponding author, Dr Birru Talabi confirms that all authors have provided the final approval of the version to be published and takes responsibility for the affirmations regarding article submission (eg, not under consideration by another journal), the integrity of the data presented, and the statements regarding compliance with institutional review board/Declaration of Helsinki requirements.

## Supporting information


**Disclosure Form**:
